# Uptake of HIV testing and its correlates among sexually experienced college students in Southwestern, China: a Web-Based online cross-sectional study

**DOI:** 10.1186/s12889-023-16638-z

**Published:** 2023-09-04

**Authors:** Jinfeng He, Ping Cen, Jiao Qin, Weiao Qin, Xiudong Xu, Yuanhong Yang, Jinglan Wu, Mu Li, Rongjing Zhang, Tong Luo, Zhifeng Lin, Xinju Huang, Chuanyi Ning, Hao Liang, Li Ye, Bin Xu, Bingyu Liang

**Affiliations:** 1 Guangxi Key Laboratory of AIDS Prevention and Treatment, School of Public Health, Guangxi Medical University, Nanning, 530021 Guangxi China; 2 Collaborative Innovation Centre of Regenerative Medicine and Medical Bioresource Development and Application Co-Constructed By the Province and Ministry, Life Science Institute, Guangxi Medical University, Nanning, 530021 Guangxi China; 3 Nanning Center for Disease Prevention and Control, 55, Xiangzhu Avenue, Nanning, 530023 China; 4 Nursing College, Guangxi Medical University, No. 8 Shuangyong Road, Nanning, 530021 Guangxi China

**Keywords:** HIV, HIV testing, College sexually experienced students, Sexual education, Anal sex

## Abstract

**Background:**

The prevalence of human immunodeficiency virus (HIV) is becoming more common among college students in China. However, latest data on the prevalence and correlates of HIV testing among sexually experienced college students is rarely.

**Methods:**

An online survey was conducted among college students aged 18 years or older using multistage stratified cluster sampling from 16 colleges. Data on socio-demographic, HIV testing, HIV-related awareness, attitudes, sexual education and behaviors were collected. Propensity score matching (PSM) and logistic regression model were used to identify factors associated with HIV testing.

**Result:**

A total of 108,987 students participated the survey, of which 13,201 sexually experienced college students were included in this study. 1,939 (14.69%) college students with sexual experience reported uptake of HIV testing in the preceding year. The uptake of HIV testing increased for college students with a rising HIV knowledge score and sexual health knowledge. Being awareness of HIV-related knowledge (aOR = 1.15, 95%CI: 1.01–1.30), accepting one-night stands (aOR = 1.16, 95%CI:1.03–1.32), obtaining satisfactory sexual interpretation from parent(s) (aOR = 1.24, 95%CI: 1.07–1.43), ever had unintended pregnancy (aOR = 1.78, 95%CI: 1.32–2.38), ever had received HIV-related preventive service(s) (aOR = 1.37, 95%CI: 1.10–1.70), ever had participated HIV-related preventive services (aOR = 3.76, 95%CI: 2.99–4.75) and ever had anal sex (aOR = 2.66, 95%CI: 2.11–3.34) were positively associated with uptake of HIV testing. However, accepting premarital sex (aOR = 0.76, 95%CI: 0.66–0.88), accepting cohabitation (aOR = 0.75, 95%CI: 0.61–0.92), occasionally discussing sex with parent(s) (aOR = 0.68, 95%CI: 0.50–0.91), and being with moderate satisfaction of school sex courses (aOR = 0.74, 95%CI: 0.58–0.95) were negatively associated with uptake of HIV testing.

**Conclusion:**

The prevalence of HIV testing was relatively low. Participation in HIV-related services and high-risk sexual behaviors were important enablers for testing. Improving sex education for students, increasing HIV preventive services on campus, and improving family sex education are necessary to increase HIV testing among college sexually experienced students.

**Supplementary Information:**

The online version contains supplementary material available at 10.1186/s12889-023-16638-z.

## Background

Human immunodeficiency virus (HIV) testing is a key policy response to the HIV/Acquired Immune Deficiency Syndrome epidemic (AIDS), has been promoted as a primary prevention strategy and an entry point for HIV care, remaining the main strategy to dam the rising HIV infection [1]. Early HIV testing and immediate treatment of HIV-infected individuals could dramatically reduce or even eliminate HIV transmission. In 2018, less than 70% people living with HIV/AIDS (PLWHA) were diagnosed and aware of their infection status [2]. There were 1.045 million PLWHA reported in China by October 2020 [3], indicating that HIV testing still needs great efforts to improve.

HIV late diagnosis poses challenges of less favorable individual treatment, high risk of HIV transmission and suboptimal public health prevention [4, 5]. Generally, advanced patients do not receive HIV tested until symptoms appear [4]. In addition, late diagnosis increases the number of unaware patients and HIV-related deaths [6, 7]. Increasing uptake of HIV testing may be an effect way to reduce late HIV diagnosis. Primary and secondary prevention and treatment of HIV among young people, including key populations of vulnerable youth, require HIV testing [8], which helps achieve the 95–95-95 target [9].

These studies were conducted among young students and found that HIV testing was primarily associated with willingness to test and with high-risk behaviors, such as anal sex [10], having three or more sexual partners [11], and unprotected sex [10]. College students with sexual experience are the most at risk for HIV infection. The existing studies mainly focused on the correlates of HIV testing willingness, which is significantly associated with behavioral factors (e.g., high-risk behaviors or unprotected sex) [12, 13]. There was limited data on HIV testing and its correlates among sexually experienced college students in recent years. Therefore, it is of great importance to assess the uptake of HIV testing and its correlates to increase HIV testing.

Guangxi, located in the Southwest of China, has a high prevalence of HIV/AIDS [14, 15]. HIV incidence across Guangxi had remained relatively stable at a high level. The number of newly diagnosed college students with HIV has showed an annual growth rate from 30 to 50% over the past several years [16]. A large proportion of newly diagnosed patients with HIV presented with late presentation in Guangxi, and HIV cases among Guangxi youngly students are increasing annually, from 2010 to 2017 [17]. The increased HIV prevalence has frequently been reported among college students [8, 16–20]. This trend is due to several reasons: being sexually active [16], being open to casual sex [16, 20], having multiple partners [20], not insisting on condom use, and having sex after using alcohol [8]. If college students living with HIV are in the early stage of disease progression and are undiagnosed or untreated, they would be susceptible to transmit HIV to other unprotected and vulnerable individuals. Therefore, college students were one of the major subgroups for HIV prevention [21].

We conducted an online cross-sectional survey among college students from 16 colleges in Nanning, Guangxi, using a stratified cluster sampling. This study aims to examine the uptake of HIV testing, to assess how this prevalence may vary by gender, age, grade and stage of study, and to determine the correlates of HIV testing among sexually experienced students in Guangxi. This will contribute immensely to the development and implementation of HIV testing promotion interventions for college students.

## Methods

### Study setting and participants

A cross-sectional survey was conducted by the Nanning Centre for Disease Control and Prevention using a combination of stratified sampling and cluster sampling among college students from 16 colleges in Nanning City from October 2020 to March 2021. We did repeat pre-surveys before the questionnaire was formally administered. The Cronbach’s alpha of the questionnaire was 0.751 and the Measure Sampling Adequacy (MSA) was 0.82.

The inclusion criteria for college students were: (1) aged 18–40 years; (2) agreed to complete the questionnaire and provided informed consent online; (3) were college students; (4) were able to read in Chinese and complete the questionnaire independently.

The prevalence of sexual behaviors among Chinese college students ranged from 9.0% to 13.4% [22–24]. We assumed that the prevalence of sexual behaviors among college students in Guangxi was 11.0%.

$$n=\left(\frac{Z_{1-\alpha/2}}\delta\right)^2\times p\times(1-p)$$

α was set to 0.05, ${\mathrm{Z}}_{1-\mathrm{\alpha }/2}$ represents the standard normal distribution bound at an alpha of 0.05. δ represents the permissible error taken as 0.011. A sample size of *n* = 3198 was calculated by using the Power Analysis and Sample Size Software version 21.0.3, taking into account a 15% drop-out rate, a minimum of 3,763 participants were required.

This study employed a cross-sectional multi-stage cluster sampling design, which involved a stratified sampling process with three stages. At the first stage, a random selection of sixteen colleges was made from a total of 35 colleges. At the second stage, four college departments were randomly chosen from each of the sixteen selected colleges. At the third stage, the students from each of the selected colleges were categorized into six strata based on their grade levels, including grades 1 to 5, as well as graduate students. The sampling process entailed selecting five classes from grades 1 to 3, two classes from grades 4 to 5, and one class from the graduate students, ensuring that the proportion of students selected from each college ranged between 5–10%.

We utilized the online data collection platform, Questionnaire Star Survey (https://www.wjx.cn/), to create the electronic versions of the questionnaires. Subsequently, the survey platform generated a two-dimensional code and link, which were then disseminated to college students by their tutors through Wechat or QQ groups. To participate in the study, college students had to agree and complete the questionnaires online, either by scanning the two-dimensional code or clicking the link on their mobile phones. The survey continued until the number of eligible records met the predetermined sample size requirement. Each survey record was assigned a unique number for participant identification purposes. The survey system implemented measures to prevent duplicate responses by allowing only one submission per electronic device. No incentives were offered for participation, and the online survey system recorded the time and location of the questionnaire submissions.

### Questionnaire design

#### HIV testing

Uptake of HIV testing was assessed via the question “Have you ever been tested for HIV before?” with response options Yes, No, or Don’t Know. We determined that those who answered “Yes” had been tested for HIV.

#### Sociodemographic characteristics and HIV/Sexual health-related knowledge

The sociodemographic characteristics variables considered in this study included gender, age, marital status, registered residence, ethnicity, stage of study, grade, social relationship (single or not), sex orientation, love experience, and sexual needs. HIV-related knowledge was assessed using a scale developed by the Chinese National Center for AIDS/STDs Control and Prevention [25], comprising eight questions, and those who answered six or more correctly were considered aware. Additionally, sexual health knowledge was assessed using eight self-designed questions, including knowledge of pregnancy and sexually transmitted infections and diseases. Similarly, those who answered six or more questions correctly were considered aware.

#### Sexual attitudes and sex education

Attitudes toward sex were measured by asking participants three questions about their attitudes toward premarital sex, premarital cohabitation, and one-night stands. Each response option was responded to by “acceptable”, and “unacceptable”. We also assessed the willingness to have a love affair.

Sex education included school sex education and family sex education. School sex education was measured the stage of participation in school sex education, and the satisfaction of school sex education. Family sex education was measured by variables such as parents' ability to answer sex-related questions, frequency of discussion of sex-related matters with parents, and family sexual perceptions.

#### Behavioral characteristics and HIV/AIDS prevention service use

Behavioral characteristics comprised the following: having had sex, having sex with casual partners, having commercial sex, having anal sex, having unintended pregnancy (She or his sexual partner), and having drug use in the recent year.

HIV/AIDS prevention service use was measured by three questions: (1) having ever received any education about HIV/AIDS knowledge in the last year; (2) participated in HIV/AIDS prevention volunteer activities in the last year; (3) proactively searched for sexual health knowledge online.

#### Data analysis

The purpose of the study was to explore the correlates for uptake of HIV testing among sexually experienced college students in Guangxi. We measured the definition of sexually experienced college students through the question, ‘Have you ever had sexual intercourse?’ if the response was ‘Yes’, the student was considered to have had sexual intercourse and was included in the study, while the response was ‘No’, they were excluded from the analysis.

First, descriptive statistics were used to describe the difference of HIV testing in socio-demographic, HIV-related knowledge, and sexual health related knowledge by EXCEL (Microsoft Corporation, Redmond, USA). To minimize the differences in socio-demographic characteristics between the two groups and to improve the efficiency of the study, we conducted PSM. PSM by MatchIt package version 4.4.0 in R version 4.1.3 was performed on socio-demographic characteristic, including gender, age, marital status, registered residence, ethnicity, stage of study, grade, social relationship (single or not), sex orientation, love affair experience and sexual needs, to keep the comparability of two groups of whether tested for HIV or not. Second, univariate and multivariate logistic regression were used to identify the correlates of uptake of HIV testing by R software (version 4.1.3). Figure was plotted by using the ggplot2 package (version 3.4.0) of R software. Factors significant at the 0.05 level in univariate analysis were entered into the multivariable model. All variables were analyzed for collinearity by bivariate correlation analysis, and none of the variance inflation factor (VIF) exceeded 7.5 and all Tolerances values exceeded 0.1, indicating low collinearity. The PSM ratio was set to 1:4 and the caliper to 0.05. Therefore, we obtained 8884 respondents included in the model analysis.

#### Ethical

This study has been reviewed and approved by the Ethics Committee of Guangxi Medical University (no.2019-SB-088). All respondents were aware of the content and purpose of this study and agreed to provide informed consent online before this survey.

## Results

### Overview of survey data collected

In total, 108,987 students participated the survey. After filtering out those who answered negatively to the question “Have you ever had sex,” a total of 15,098 sexually experienced students remained. Subsequently, 1897 questionnaires with missing items or significant logical errors were excluded, leaving 13,201 valid questionnaires. Logical errors were identified based on inconsistent age and stage of study, inaccurate age of first sexual intercourse, and erroneous marital status reported by single students who indicated cohabitation or marriage. “Inaccurate age of first sexual intercourse” meant that the age entered was older than the age at the moment of the survey.

### Socio-demographic characteristic

Table 1 presents the demographic characteristics of the participants. A total of 13,201 respondents were included in this study. Among them, 1,939 (14.69%) reported that they had been tested for HIV, and 16 cases tested positive for the antibody. The majority of the respondents, 13,066 (98.98%), were aged between 18 and 28, with a median age (interquartile range) of 21 (19–23) years. Males accounted for 61.35% (8,099), and 96.74% (12,770) of the participants were unmarried. About half of the participants, 7,624 (57.75%), were of Han ethnicity, while 4,475 (33.90%) were of Zhuang ethnicity. The grades were categorized into three levels: high grades, low grades, and postgraduate and above, with most of them were in the low grades (93.65%, 9,723). The stages of study were mainly college (54.00%, 7,134) and undergraduate (43.00%, 5,683).

**Table 1:** Demographic characteristic of sexually experienced college students (*N* = 13201)

Variable	Total (*n* = 13201)	HIV testing	
		**No (%)**	**Yes (%)**
**HIV testing**		11262(85.31)	1939(14.69)
**Marriage**			
Unmarried	12770(96.74)	10962(83.04)	1808(13.70)
Married	431(3.26)	300(2.27)	131(0.99)
**Gender**			
Male	8099(61.35)	6715(50.87)	1384(10.48)
Female	5102(38.65)	4547(34.44)	555(4.20)
**Region**			
Guangxi	11618(88.01)	9954(75.40)	1664(12.61)
Outside Guangxi	1583(11.99)	1308(9.91)	275(2.08)
**Age**			
(18, 28)	13066(98.98)	11191(84.77)	1875(14.20)
(28,40)	135(1.02)	71(0.54)	64(0.48)
**Nation**			
Zhuang	4475(33.90)	3897(29.52)	578(4.38)
Han	7624(57.75)	6429(48.70)	1195(9.05)
Other	1102(8.35)	936(7.09)	166(1.26)
**Grade**			
Low grades (1–2)	9723(73.65)	8190(62.04)	1533(11.61)
High grades (3–5)	3307(25.05)	2937(22.25)	370(2.80)
Postgraduate and above	171(1.30)	135(1.02)	36(0.27)
**Stage of study**			
Junior college and below	7260(55.00)	6139(46.51)	1123(8.49)
Undergraduate and above	5941(45.00)	5123(38.81)	818(6.20)
**Sex orientation**			
Homosexual	1483(11.23)	1129(8.55)	354(2.68)
Heterosexual	11718(88.77)	10133(76.76)	1585(12.01)
**Single or not**			
Not	7397(56.03)	6494(49.19)	903(6.84)
Yes	5804(43.97)	4768(36.12)	1036(7.85)
**Love experience**			
No	934(7.08)	769(5.83)	165(1.25)
Yes	12267(92.92)	10493(79.49)	1774(13.44)
**Sexual needs**			
Rarely	11375(86.17)	9715(73.59)	1660(12.57)
Frequently	1826(13.83)	1547(11.72)	279(2.11)
**HIV-related knowledge**			
Unaware	3,947(29.90)	3,465(26.25)	482(3.65)
Aware	9254(70.10)	7797(59.06)	1457(11.04)
**Sexual health related knowledge**			
Unaware	10908(82.63)	9558(72.40)	1350(10.23)
Aware	2293(17.37)	1704(12.91)	589(4.46)

### HIV-related knowledge and sexual health related knowledge

Most of the respondents (70.10%, 9,254) were aware of HIV-related knowledge, while 10,908 (82.63%) participants were unaware of sexual health knowledge (Table 1). Table 2 displays the response of HIV and sexual health-related questions. Only two HIV-related questions, the questions of “Consistent and correct use of condoms can reduce the risk of HIV infection” and “After engaging in high-risk behaviors, such as needle sharing, drug use, or unsafe sex, should people actively seek HIV testing and counseling?”, had a correct response rate above 95.00%. The lowest percentage of correct answers was for the question of “The main HIV transmission route among students in China is homosexual, followed by heterosexual, right?”, which asked about the current state of the HIV epidemic among young students in China and the main mode of transmission. Only 84.10% of participants answered this question correctly. The proportion of participants who reported ever being tested for HIV was over 12% for each correctly answered HIV-related question.

**Table 2:** Response of HIV-related and sexual health knowledge and uptake of HIV testing

Variable	Response	Total (%)	HIV testing
			Yes (%)
**HIV-related knowledge**			
AIDS is a serious and incurable infectious disease	Incorrect	1497 (11.34)	200 (13.36)
	Correct	11704 (88.66)	1739 (14.87)
The main HIV transmission route among students in China is homosexual, followed by heterosexual, right?	Incorrect	2099 (15.90)	249 (11.86)
	Correct	11102 (84.10)	1690 (15.22)
A person infected with HIV can be identified by appearance?	Incorrect	1660 (12.57)	410 (24.70)
	Correct	11541 (87.43)	1529 (13.25)
Daily contacts can transmit HIV	Incorrect	1569 (11.89)	376 (23.96)
	Correct	11632 (88.11)	1563 (13.44)
Consistent and correct use of condoms can reduce the risk of HIV infection	Incorrect	601 (4.55)	106 (17.64)
	Correct	**12600 (95.45)**	**1833 (14.55)**
The use of new drugs (Such as Methamphetamine, Ecstasy, Ketamine, etc.) increases the risk of HIV infection	Incorrect	1091 (8.26)	169 (15.49)
	Correct	**12110 (91.74)**	**1770 (14.62)**
After engaging in high-risk behaviors, such as needle sharing, drug use, or unsafe sex, should people actively seek HIV testing and counseling?	Incorrect	469 (3.55)	83 (17.70)
	Correct	**12732 (96.45)**	**1856 (14.58)**
The rights of people living with HIV (Such as marriage / employment / schooling) are protected by Chinese law	Incorrect	1462 (11.07)	184(12.59)
	Correct	11739 (88.93)	1755 (14.95)
**Sexual health knowledge**			
Having sex before menstruation (14 days) is likely to get pregnant	Incorrect	4350 (32.95)	532 (12.23)
	Correct	**8851 (67.05)**	**1407 (15.90)**
Sperm can survive in a woman’s uterus or vagina for about 7 days	Incorrect	4926 (37.32)	607 (12.32)
	Correct	8275 (62.68)	1332 (16.10)
If ejaculation outside the body can effectively prevent pregnancy?	Incorrect	3824 (28.97)	809 (21.16)
	Correct	**9377 (71.03)**	**1130 (12.05)**
Having sex in a safe period can effectively avoid pregnancy	Incorrect	8408 (63.69)	1050 (12.49)
	Correct	4793 (36.31)	889 (18.55)
Mosquito bites can transmit HIV	Incorrect	9714 (73.59)	1268 (13.05)
	Correct	3487 (26.41)	671 (19.24)
Genital herpes is a sexually transmitted disease	Incorrect	3228 (24.45)	370 (11.46)
	Correct	**9973 (75.55)**	**1569 (15.73)**
After pregnancy, menstruation will continue for two or three months	Incorrect	9339 (70.74)	1108 (11.85)
	Correct	3862 (29.26)	831 (21.52)
Painless abortion is safer than ordinary abortion	Incorrect	8696 (65.87)	1049 (12.06)
	Correct	4505 (34.13)	890 (19.76)

None of the sexual health-related questions had a correct response rate above 95.00%. The questions with the highest percentage of correct responses were the questions of “If ejaculation outside the body can effectively prevent pregnancy? and “Genital herpes is a sexually transmitted disease”, both of which had a correct response rate of more than 70%. Furthermore, increasing HIV knowledge scores and sexual health knowledge were associated with increased rates of HIV testing among college students.

### Sexual attitudes and sex education

Regarding sexual attitudes, 73.23% (9667/13201) of the participants accepted premarital sex, 87.43% of them (11,542/13201) accepted cohabitation, and 47.62% of them (6,286/13201) accepted one-night stands. Approximately half (50.45%, 6660) of the students held a casual attitude towards love affairs. Among them, 11.49%, 12.30%, and 15.08% had been HIV tested, respectively (Additional file 2, S. Figure 2).

The sex education section covers family and school education. In family sex education, 44.69% (5,899) of the participants lacked sexual health discussions with parents, 11.89% (1,569) of them received moderate responses, and only 3.31% (437) frequently discussed sex. Majority (74.17%, 9791) never discussed sex, while 51.30% (6772) considered their family's attitude towards sex as conservative and 19.58% (2585) as enlightened. For the sex education, 44.20% (5835/13201) had sex education in middle school, while 26.06% of them participated sex education in primary school. 37.25% (4917/13201) of them were moderately satisfied with sex education, and 56.38% (7443/13201) were satisfied with school sex education courses (S. Figure 2).

### Behavioral characteristics and HIV/AIDS prevention service use

In terms of behaviors characteristics, the majority (93.24%, 12308) of participants abstained from engaging in anal sex over the past year. Moreover, a substantial majority (70.52%, 9309) participated in sexual activity in past year. Additionally, a majority (54.00%, 7128) of them did not reside with a heterosexual friend, and most of them (86.01%, 11354) did not have sexual partners. Furthermore, an majority (94.47%, 12,471) of them refrained from involvement in commercial sex within the recent year. A fraction of students (5.57%, 735/13201) or their partner experienced unintended pregnancies, while only a minor percentage (3.16%, 417/13201) reported drug abuse (S. Figure 2).

About 81.83% (10802/13201) of the participants have received themselves of HIV-related prevention services, while an additional 78.83% (10407/13201) have participated in such services. Furthermore, a majority (76.96%, 10159) of them frequently access sexual health information online (S. Figure 2).

### Correlates of HIV testing

**Fig. 1:**
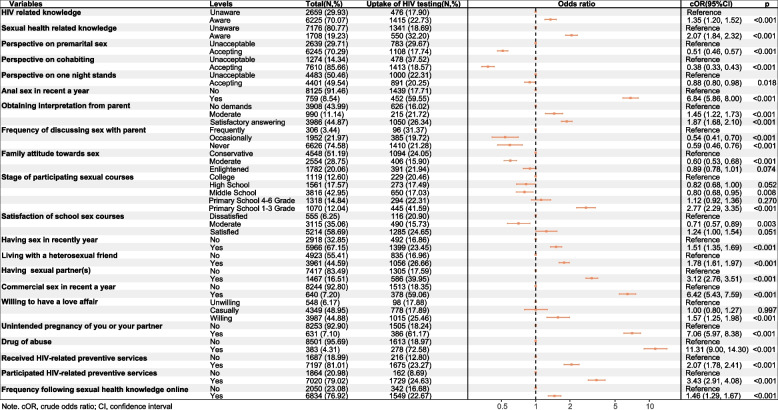
Univariate analysis for correlates of HIV testing among sexually experienced college students after PSM (*n* = 8884)

**Fig. 2:**
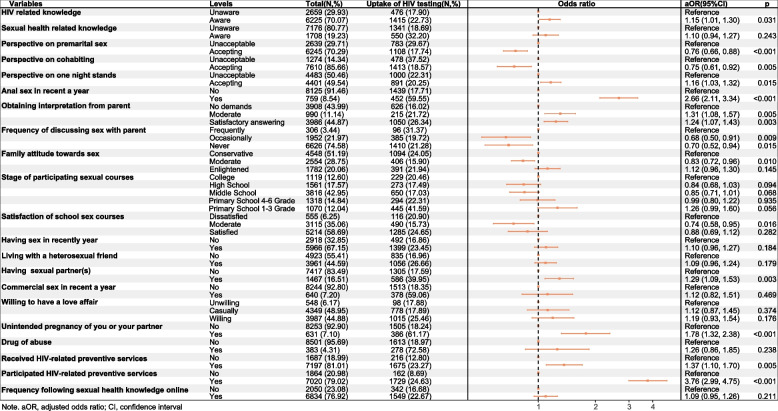
Multivariate analysis for correlates of HIV testing among sexually experienced college students after PSM (*n* = 8884)

Figures 1 and 2 illustrate the associations of HIV testing uptake. Prior to PSM, the variable for one-night stand perspectives showed no significant variation in the univariate analysis; however, it demonstrated a significant difference after PSM. Conversely, the remaining variables exhibited significant differences both before and after PSM (S. Figure 2 and Fig. 1). Therefore, we included all variables in the multivariate analysis, which remained consistent before and after PSM (Additional file 2, S. Figure 3 and Fig. 2). To enhance the study efficiency and mitigate the impact of socio-demographic attributes, we conducted the multivariate analysis model after PSM. In the multivariate analysis model after PSM, students who were knowledgeable about HIV-related information (aOR 1.15,95%CI 1.01–1.30) were more likely to undergo HIV testing than those who were not. Regarding sexual attitudes, students who accepted premarital sex (aOR 0.76,95%CI 0.66–0.88) or cohabitation (aOR 0.75,95%CI 0.61–0.92) were less likely to undergo HIV testing. Participants who accepted one-night stands (aOR 1.16,95%CI1.03–1.32) were more likely to undergo HIV testing than those who refused. With regard to students' family sex education, those sexually experienced students whose parents could partially (aOR 1.31,95%CI 1.08–1.57) and satisfactorily (aOR 1.24,95%CI 1.07–1.43) answer their child's sex questions were more likely to undergo HIV testing than those who had no demand or no response from their parents. Participants who evaluated their family's perception of sex as moderate (aOR 0.83,95%CI 0.72–0.96) were less likely to undergo HIV testing than those evaluated as conservative. Students who were moderately (aOR 0.74,95%CI 0.58–0.95) satisfied with school sex education were less likely to test for HIV than those who were dissatisfied with school sex education. With regard to sexual behaviors, students who ever had casual partner(s) (aOR 1.29, 95%CI 1.09–1.53), ever engaged in anal sex (aOR 2.66, 95%CI 2.11–3.34), and ever had experienced unintended pregnancy (aOR 1.78,95%CI 1.32–2.38) were more likely to undergo HIV testing. In addition, students who had received (aOR 1.37, 95%CI 1.10–1.70) or participated (aOR 3.76,95%CI 2.99,4.75) in HIV-related services were more likely to undergo HIV testing.

## Discussion

This study utilized a large sample size to investigate the uptake and correlates of HIV testing among sexually experienced college students in a region of Southwestern China with a high HIV burden. HIV testing plays a crucial role in reducing the HIV/AIDS epidemic, especially among young adults. Our findings indicate a low prevalence of HIV testing among the participants, and we have demonstrated that certain risk sexual behaviors such as ever having anal sex, ever having casual sex, and ever having unintended pregnancy (either in oneself or their partners), as well as receiving or participating in HIV-related preventive services, having correct HIV-related knowledge, accepting one-night stands, and receiving sex education and interpretation from parents, were positively associated with HIV testing.

In this study, the prevalence of uptake HIV testing was much lower than that of U.S. high school [26], Italy [27], and male students [28], while it was higher than that reported in other provinces and those from large cities in China [29–33]. This finding suggested that HIV testing is not common among students in China. There are several explanations for this low uptake of HIV testing. First, some students believed there was no need to be tested for HIV as they only had sex with steady partners, consistent with other findings in this study that students who exhibited open attitudes to premarital sex and cohabited sex were less likely to be tested for HIV. Second, in China, the community HIV/AIDS awareness campaigns targeting general population do not widely emphasize HIV testing [34]. Third, prejudices and discrimination against HIV/AIDS in general population is a global barrier of HIV testing, as some students worry about others’ attitudes, and worry about a positive result [32]. This low uptake of testing deserves consideration, as a national study has reported an annual increase in the number of new HIV diagnoses in the youth population [35], and nearly 40% of new HIV infections are transmitted by those who are unaware of their HIV diagnosis [36].Given the low uptake of HIV testing among sexually experienced students, innovate HIV prevention programs specifically designed to increase the accessible, convenience and private HIV testing channels on campus is warranted.

Alarmingly, the proportion of correct responses to HIV-related knowledge was as low as a national survey [32], and far lower than the target HIV knowledge rate among college students at 95% in the Implementation Plan for the Containment and Control of HIV/AIDS (2019–2022) [37]. However, knowledge of HIV transmission was higher than Italy [38]. And knowledge of HIV transmission and HIV prevention was higher than South Carolina [39], but lower than universities in the southeastern United States [40]. We found that the uptake of HIV testing increased for college students with a rising HIV-related knowledge and sexual health knowledge score. In China, the low level of HIV-related knowledge and sexual health knowledge among university students may be due to the influence of traditional mentality [22, 41], as well as parents' perceptions and attitudes towards sexuality [42], and lack of effectively sexual courses in some Chinese colleges [43]. Moreover, college students with sufficient HIV-related knowledge were more likely to test for HIV than those who with insufficient HIV-related knowledge, are consistent with previous studies [44–47]. Poor knowledge may lead to failed to apply for preventive measures to reduce HIV transmission at the individual and community levels [27]. Sufficient knowledge of HIV contributes to the uptake of HIV testing and is a positive motivation to consistent condom use [38, 40]. HIV-related knowledge awareness is crucial for reducing the risk of infection, promoting willingness to undergo HIV testing, and improving HIV detection and treatment, making it an essential part of HIV testing. Thus, more effort is needed to expand and strengthen HIV related advocacy in students, leading to improvements in HIV testing.

We noted that high-risk sexual behaviors, including past anal sex, casual sex, and unintended pregnancy were associated with increased uptake of HIV testing, are in line with observation from previous studies [26, 46, 48–50]. The Men who have Sex with Men (MSM) cohort has gained prominence in recent times and was duly offered HIV screening during consultation with relevant healthcare services [51, 52]. Their HIV risk perception could increase due to indulging in anal intercourse [53]. Furthermore, the incidence of casual sex and HIV risk perception exhibited a direct correlation, thereby augmenting the number of individuals seeking HIV testing after casual sexual encounters [52, 53]. Additionally, the majority of unintended pregnancy stem from unprotected sexual activity, and routine testing for HIV and other sexually transmitted infections is typically performed before abortion procedures [54, 55]. High-risk sexual behavior directly affects the perception of risk and contributes to the need for HIV testing [48, 56–58]. Therefore, it is significant that more attention should be given to sexual health education and to increase the HIV/STIs infection risk perception in colleges to reduce high-risk sexual behavior and promote HIV/STI testing.

With the widespread use of social media, college students tended to be open-minded about the sexual attitudes and sexual behaviors, and were accepting premarital sex, cohabitation, and other sex-related practices [59, 60]. Although, the prevalence of STIs in college students is on the rise, the uptake of HIV/STIs testing is disappointing among the young students [44]. Some young students considered that it was not possible to contract HIV with steady partners [61], which prevent them from HIV testing. Our study showed that college students who accepted cohabitation and premarital sex were less likely to be tested HIV than those with contrary attitudes. Cohabitation and premarital sex which involve steady partners, might reduce the risk perception of HIV infection, and thus reduces the likelihood of testing [48, 62]. However, individuals who accepted one-night stand were more likely to be tested for HIV than those with opposite view. The unstable relationship in one-night stands and unawareness of partner’s HIV status heightened their perception of HIV risk [48], and the uptake of HIV testing. Therefore, increasing students’ risk perception of HIV infection and guiding to safe sex would be helpful for HIV prevention.

In this study, the satisfaction of sex education was significantly associated with HIV testing. Sex education is one of the essential ways to form positive sex attitudes and safe sex behaviors. However, as sexuality is a taboo and embarrassing subject in China, sex education is inadequate or even absent for a long time [59]. The common problem with sex education in China is that teachers did not receive special training before dissemination of sex health knowledge to students [59]. In 2020, almost half of the freshmen in college did not receive sex education [63]. At present, the social media has become the main source the main sources of sex health knowledge. However, the sex information online lacks sufficient regulation from law enforcement department, and students lack judgment [64]. As a result, students may get the incorrect sex health information. Sufficient and correct knowledge of sexual health is fundamental to the prevention of STDs. The inadequacy of sex education calls for a need to plan and implement satisfactory, comprehensive, and standardized sex education in the overall education system in China.

We noted that sexually experienced college students who had ever received satisfactory interpretations about sex from their parents, as well as those students who reported more frequency of discussion about sex with parents, were more likely to take HIV testing. This finding indicated the importance of home-based sex education for the sexual health of college students. Parents play a primary role in educating sex to their children, and young people often want to learn about sex health knowledge from their parents [65]. However, a low proportion of parents involved in sex education [42], and only one-third of the parents had talked to their children comfortably about sex [42]. Thus, it is recommended to be a collaborative program involves families and colleges on sex education.

We also found that students who had ever received and participated in HIV-related services were more likely to undergo HIV testing. The dissemination of health science knowledge is like a social vaccine that can effectively reduce the prevalence of diseases [66]. Students who received or participated in HIV-related services may be more aware of the current serious HIV epidemic situation and HIV infection risks. Some HIV/AIDS related educational services may even offer HIV testing directly to participating students [67]. In areas with elevated HIV prevalence, there is a great demand for HIV-related social services, which are one of the most urgent needs [68]. We recommend that HIV services, including HIV testing, HIV education, and sex education, should be a universal service for colleges. For example, automated HIV urine testing services or school nurses should provide testing services, especially in Guangxi, where the HIV epidemic is severe.

### Limitations

Our study has several limitations. Firstly, this survey used self-reported items, which are susceptible to recall and social desirability bias. This could potentially lead to an overestimation of actual testing behavior or an underestimation of sexually risky behavior. Secondly, an online self-administered questionnaire was used to collect data, so the reliability of the data depends on the sincerity and responsibility of the participants. Thirdly, although this study had a large sample size, it was confined to a single major metropolitan area, and the findings cannot be extrapolated to other regions. Fourthly, due to the constraints of the cross-sectional study, a causal link between HIV testing and associated variables could not be established. Finally, we were unable to achieve complete randomization sampling during the implementation of the online survey, and there was no fixed number of sampled classes for each grade. Instead, it was a range value, making it impossible to calculate exact weights. To reduce the impact of sampling, we increased the sample size and improved the testing efficiency statistically by matching. Due to the large proportion of missing values for the question about condom use, it was not included in the analysis in this study. We avoided this issue through multiple questions in the ongoing survey. Despite these limitations, the findings were credible and could bridge gaps in the data and correlates of HIV testing among sexually experienced students in Guangxi. Specifically, we quantified the proportion of sexually experienced college students who had undergone HIV testing, analyzed variations in HIV testing uptake across sex-related behaviors, attitudes, education, knowledge, and services, and explored the reasons for the low prevalence of HIV testing uptake.

## Conclusion

This study has demonstrated a low prevalence of HIV testing uptake among sexually active college students. The findings indicate that college students with appropriate HIV-related knowledge, high-risk sexual behavior, parental involvement in sex education, and participation in HIV-related preventive services are more likely to undergo HIV testing. Based on these findings, we recommend comprehensive and high-quality sex education programs for college students, families, and school healthcare departments. It is imperative not only to provide HIV prevention services on campus but also to encourage college students to participate in these activities to enhance their HIV-related knowledge and improve the accessibility and effectiveness of HIV testing. Moreover, rapid and innovative HIV testing services like urine screening and saliva testing programs should be established on college campuses to ensure prompt access to HIV testing.

### Supplementary Information

**Additional file 1. **

**Additional file 2: S. Figure 1.** The proportion of correct response and uptake of HIV testing with HIV and sexual health related knowledge. **S. Figure 2.** Univariate analysis for correlates of HIV testing among sexually experienced college students before PSM (*n*=13201). **S. Figure 3.** Multivariate analysis for correlates of HIV testing among sexually experienced college students before PSM (*n*=13201).

## Data Availability

The datasets generated and/or analyzed during the current study are not publicly available because of ethical and legal reasons but are available from the corresponding author Bingyu Liang on reasonable request.
