# Supplementary material for: Uptake of HIV testing and its correlates among sexually experienced college students in Southwestern, China: a Web-Based online cross-sectional study
Source: BMC Public Health. 2023 Sep 4;23:1702. doi: 10.1186/s12889-023-16638-z (PMC10476433; doi:10.1186/s12889-023-16638-z)
Supplement: Supplementary file 1 — Additional file 1. [file 12889_2023_16638_MOESM1_ESM.docx]

**Health and behavioral status questionnaire of young students**

**Introduction**

Hello boys and girls, we are conducting a survey about the health knowledge and sexual behavioral among college students, please take a few minutes to complete this questionnaire according to your actual situation. This survey is anonymous, and all data will be used for statistical analysis only, so please feel free to finish it. We hope you can support our work. Thank you very much!

**Would you like to participate in this survey?**

①Yes ②No (End of survey)

**A. Sociodemographic characteristics**

**A01 Gender**

①Male ②Female

**A02 Age: ______years**

**A03 Marriage status**

①Single ②Married ③Cohabiting ④Divorced/Widowed

**A04 Registered residence**

1. Guangxi ②Outside Guangxi

**A05 Ethnicity**

①Zhuang ②Han ③Other

**A06 Grade**

①Freshman ②Sophomore ③Junior ④Senior ⑤Graduate

**A07 Stage of study**

①Professional college ②Bachelor ③Master

**A08 Sex orientation**

①Heterosexual ②Bisexual ③ Homosexual ④Asexual ⑤Pansexual ⑥Other:______

**A09 Are you currently single？**

1. Yes ②No

**A10 Are you have Sexual needs recently?**

1. Yes ②No

**Q. HIV-related and Sexual Health Related Knowledge**

**Q1 AIDS is a serious and incurable infectious disease.**

1. Yes ②No ③I don't know

**Q2 The main HIV transmission route among students in China is homosexual, followed by heterosexual, right?**

1. Yes ②No ③I don't know

**Q3 A person infected with HIV can be identified by appearance?**

1. Yes ②No ③I don't know

**Q4 Daily contacts can transmit HIV**

1. Yes ②No ③I don't know

**Q5 Consistent and correct use of condoms can reduce the risk of HIV infection.**

1. Yes ②No ③I don't know

**Q6 The use of new drugs (Such as Methamphetamine, Ecstasy, Ketamine, etc.) increases the risk of HIV infection.**

1. Yes ②No ③I don't know

**Q7 After engaging in high-risk behaviors, such as needle sharing, drug use, or unsafe sex, should people actively seek HIV testing and counseling?**

1. Yes ②No ③I don't know

**Q8 The rights of HIV-infected persons such as marriage/employment/schooling are protected by Chinese law.**

1. Yes ②No ③I don't know

**Q9 Having sex before menstruation (14 days) is likely to get pregnant.**

1. Yes ②No ③I don't know

**Q10 Sperm can survive in a woman's uterus or vagina for about 7 days.**

1. Yes ②No ③I don't know

**Q11 If ejaculation outside the body can effectively prevent pregnancy?**

1. Yes ②No ③I don't know

**Q12 Having sex in a safe period can effectively avoid pregnancy.**

1. Yes ②No ③I don't know

**Q13 Mosquito bites can transmit HIV.**

1. Yes ②No ③I don't know

**Q14 Genital herpes is a sexually transmitted disease.**

1. Yes ②No ③I don't know

**Q15 After pregnancy, female menstruation will continue for two or three months.**

1. Yes ②No ③I don't know

**Q16 Painless abortion is safer than ordinary abortion.**

①Yes ②No ③I don't know

**C. Sexual attitude**

**C01 What is your attitude on premarital sex?**

①Acceptable ②Neutral ③unacceptable

**C02 What is your attitude on “one night stand” or “hook up”?**

①Acceptable ②Neutral ③unacceptable

**C03 What is your attitudes on cohabiting before getting married?**

1. Acceptable ②Neutral ③unacceptable

**C04 Are you willing to have a love affair now?**

①Yes ②No

**D. Sex education**

**D01 Does your parents satisfactorily answer your questions about sex ?**

1. No demands ②Moderate ③Satisfactory answering

**D02 How often do you discuss about sex with your parents?**

1. Frequently ②Occasionally ③Frequently

**D03 How do you evaluate your family's sexual attitude**

1. Conservative ②Moderate ③Enlightened

**D04 When did you participate sexual courses in school?**

①College ②High school ③Middle school ④Primary school at 4-6 grades ⑤Primary school at 1-3 grades

**D05 Are you satisfied with your school's sex courses?**

①Dissatisfied ② Moderate ③Satisfied

**E. Behavioral characteristics**

**E01 Which of the following intimate sexual behaviors have you engaged in? (Multiple Choice)**

①Hold hands ②Hug ③Kiss ④Fondle(Non-sexual organs) ⑤Fondle(Sexual organ) ⑥Oral sex ⑦Sexual intercourse-incentive

**E02 Have you ever had sexual intercourse?**

1. Yes ②No(Skip to E18)

**E03 Have you had sexual intercourse in recent year?**

1. Yes ②No

**E04 The age of your first sexual intercourse: ______year**

**E05 Have you ever been physically forced to have sex at the first sex activity?**

1. Yes ②No ③I don't know

**E06 Did you use a condom for your first sex intercourse?**

1. Yes ②No ③Forgotten

**E07 The number of your sexual partners: ______**

**E08 Did you have sex in the past year?**

1. Yes ②No

**E09 Have you ever had unintended pregnancy (you or your partner)**

1. Yes ②No

**E10 Did you have a casual sexual partner in the last year?**

1. Yes ②No

**E11 Have you ever had commercial sex in the last year？**

1. Yes ②No

**E12 Have you ever engaged in casual sex in the last year?**

①Yes ②No (Skip to E15)

**E13** **What is the types of your casual sexual partner(s):(Multiple Choice)**

①Ex-boyfriend/girlfriend ②Classmate/friend ③Net friend ④Sex worker ⑤Stranger ⑥Relative ⑦School teacher ⑧Boss ⑨Colleague ⑩Other

**E14 What is the main reason you did not use a condom the last time you when you had casual sex? (Multiple Choice)**

①Not available ②Too expensive ③Sexual partner refused to use ④I am not willing to use ⑤Used other forms of contraception ⑥Unnecessary ⑦Forgot to use ⑧Never used previously ⑨Don't know or embarrassed to buy ⑩Others

**E15 Have you ever engaged in commercial sex in the last year?**

1. Yes ②No (Skip to E18)

**E16 Did you use a condom at the last commercial sex？**

1. Yes (Skip to E18) ②No

**E17 What is the main reason you did not use a condom the last time you had commercial sex?** **(Multiple Choice)**

①Not available ②Too expensive ③Sexual partner refused to use ④I am not willing to use ⑤Used other forms of contraception ⑥Unnecessary ⑦Forgot to use ⑧Never used previously ⑨Don't know or embarrassed to buy ⑩Others

**E18 Do you take illicit drugs?**

1. Yes ② No

**E19 Have you ever had lived with a heterosexual friend in the last year？**

①Yes ②No

**E 20 In the recent year, did you consistently use a condom when you had sex?**

① Every time ② Occasionally ③ Hardly ④ I Can't remember

**F. HIV/AIDS prevention service use**

**F01Have you ever received any HIV/AIDS knowledge education in the last year?**

①Yes ②No

**F02 Have you ever participated in HIV/AIDS prevention volunteer activities in the last year?**

①Yes ②No

**F03 Have you ever been tested for HIV in the last year?**

1. Yes ②No

**F04 Did you actively search sexual health knowledge online?**

1. Yes ②No

**This is the end of the survey, thank you for your participation!**
