# Supplementary material for: Uptake of HIV testing and its correlates among sexually experienced college students in Southwestern, China: a Web-Based online cross-sectional study
Source: BMC Public Health. 2023 Sep 4;23:1702. doi: 10.1186/s12889-023-16638-z (PMC10476433; doi:10.1186/s12889-023-16638-z)
Supplement: Supplementary file 2 — Additional file 2: S. Figure 1. The proportion of correct response and uptake of HIV testing with HIV and sexual health related knowledge. S. Figure 2. Univariate analysis for correlates of HIV testing among sexually experienced college students before PSM (n=13201). S. Figure 3. Multivariate analysis for correlates of HIV testing among sexually experienced college students before PSM (n=13201). [file 12889_2023_16638_MOESM2_ESM.docx]

S.Figure 1. The proportion of correct response and uptake of HIV testing with HIV and sexual health related knowledge.


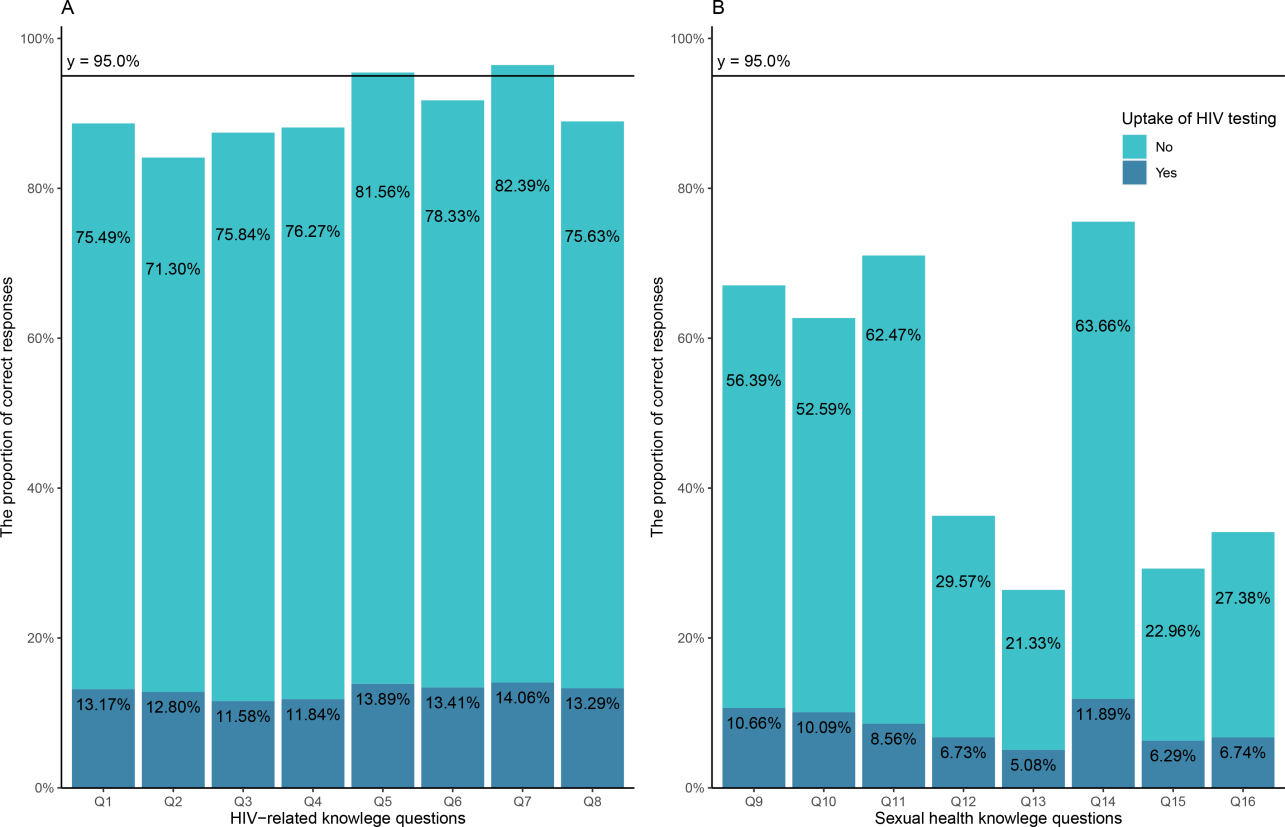


Note: A, Correct response of HIV-related knowledge(Q1-Q8) and the uptake of HIV testing of each question; B, Correct response of sexual health knowledge(Q9-Q16) and the uptake of HIV testing of each question. The two different colors indicated the uptake of HIV testing (Yes and No). (Q1: AIDS is a serious and incurable infectious disease Q2: The main HIV transmission route among students in China is homosexual, followed by heterosexual, right? Q3: A person infected with HIV can be identified by appearance?; Q4: Daily contacts can transmit HIV; Q5: Consistent and correct use of condoms can reduce the risk of HIV infection; Q6: The use of new drugs (Such as Methamphetamine, Ecstasy, Ketamine, etc.) increases the risk of HIV infection; Q7: After engaging in high-risk behaviors, such as needle sharing, drug use, or unsafe sex, should people actively seek HIV testing and counseling? Q8: The rights of HIV-infected persons such as marriage / employment / schooling are protected by Chinese law; Q9: Having sex before menstruation (14 days) is likely to get pregnant; Q10: S Sperm can survive in a woman's uterus or vagina for about 7 days; Q11: If ejaculation outside the body can effectively prevent pregnancy?; Q12: Having sex in a safe period can effectively avoid pregnancy; Q13 Mosquito bites can transmit HIV; Q14 Genital herpes is a sexually transmitted disease; Q15: After pregnancy, menstruation will continue for two or three months; Q16: Painless abortion is safer than ordinary abortion.)

S.Figure 2. Univariate analysis for correlates of HIV testing among sexually experienced college students before PSM (n=13201).


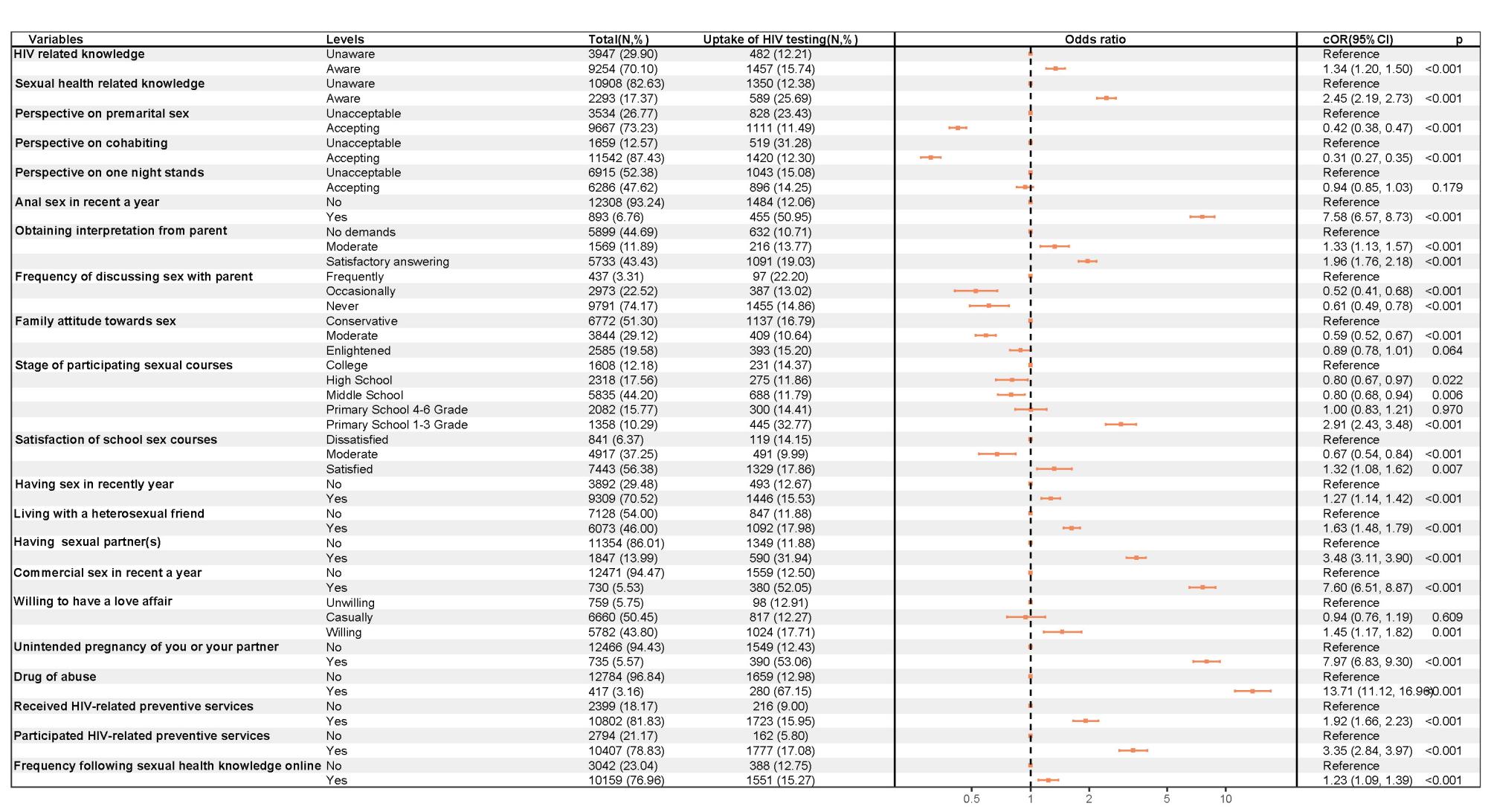


Note: aOR, cOR, crude odds ratio; CI, confidence interval

S.
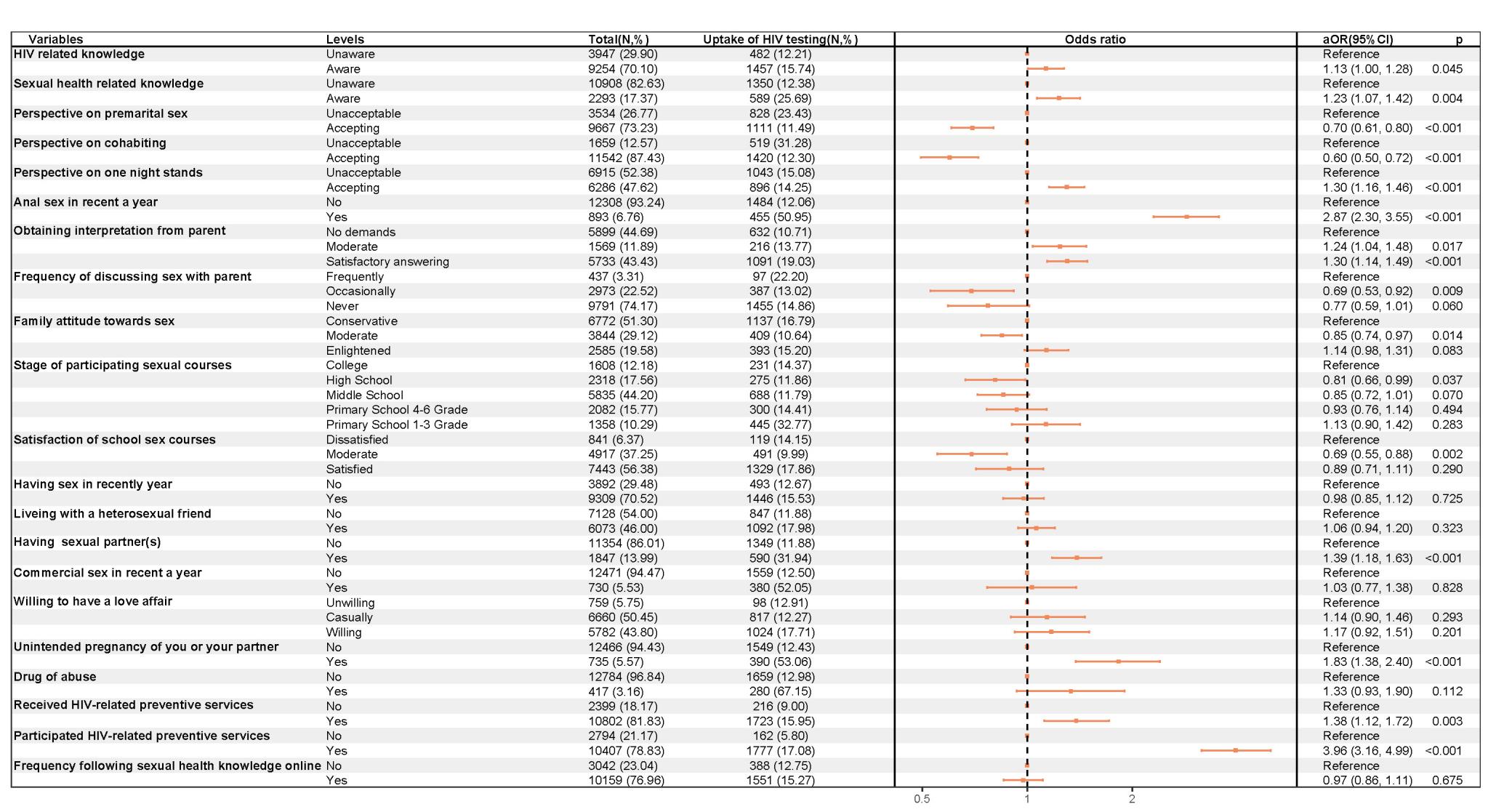
Figure 3. Multivariate analysis for correlates of HIV testing among sexually experienced college students before PSM (n=13201).

Note: aOR, adjusted odds ratio; CI, confidence interval
